# Species-specific interference exerted by the shrub *Cistus clusii* Dunal in a semi-arid Mediterranean gypsum plant community

**DOI:** 10.1186/s12898-018-0204-x

**Published:** 2018-11-29

**Authors:** Ana Foronda, Bodil K. Ehlers, Concepción L. Alados, Yolanda Pueyo

**Affiliations:** 1 Instituto Pirenaico de Ecología, CSIC, Avda. Montañana, 1005 50059 Saragossa, Spain; 2 Institut for Bioscience, Aarhus Universitet, Veljsøvej 25, 8600 Silkeborg, Denmark

**Keywords:** Aqueous extracts, Germination, Interference, Nurse plant, Survival, Richness

## Abstract

**Background:**

The gypsovag shrub *Cistus clusii* is locally dominant in semi-arid gypsum plant communities of North-Eastern Spain. This species commonly grows in species-poor patches even though it has nurse potential, suggesting interference on neighbouring species. Other *Cistus* species exert a chemically mediated interference on plant communities, suggesting that it might be a common phenomenon in this genus. This study aimed investigating whether *C. clusii* exerts chemically mediated interference on neighbouring species in gypsum plant communities. We tested in a greenhouse whether aqueous extracts from *C. clusii* leaves (L), roots (R) and a mixture of both (RL) affected germination, seedling survival, and growth of nine native species of gypsum communities, including *C. clusii* itself. We further assessed in the field richness and abundance of plants under the canopy of *C. clusii* compared to *Gypsophila struthium* (shrub with a similar architecture having a nurse role) and in open patches. Finally, we specifically assessed in the field the influence of *C. clusii* on the presence of the species tested in the greenhouse experiment.

**Results:**

Aqueous extracts from *C. clusii* (R and RL) negatively affected either germination or survival in four of nine species. In the field, richness and abundance of plants were lower under the canopy of *C. clusii* than under *G. struthium*, but higher than in open patches. Specifically, five of nine species were less frequent than expected under the canopy of *C. clusii*.

**Conclusions:**

*Cistus clusii* shows species-specific interference with neighbouring species in the community, which may be at least partially attributable to its phytotoxic activity. To our knowledge, this is the first report of species-specific interference by *C. clusii*.

**Electronic supplementary material:**

The online version of this article (10.1186/s12898-018-0204-x) contains supplementary material, which is available to authorized users.

## Background

Both interference and facilitation influence the composition and structure of plant communities [1, 2]. The interplay between interference and facilitation may be particularly important in arid and semi-arid environments, where abiotic conditions make facilitation an important process that affects local species composition [3]. Shrubs can act as nurse plants by providing under their canopy favourable microhabitats for plants and thus can harbour highly diverse microcosms [4, 5]. In early developmental stages, plants are highly vulnerable to abiotic stress and often need facilitation by nurse plants to establish [6, 7]. Once the facilitated seedlings become adults, they may exert an adverse effect on nurse plants by competitive exclusion for water, nutrients, light or space [8–11].

Some plant species produce chemical compounds that are released to the local environment through volatilisation, leaf leachates, root exudates or leaf litter decomposition [12–14]. Although these compounds can have positive effects by promoting plant growth [15] or increasing species richness [16], they are usually phytotoxic and act as selective agents that affect the performance of other species negatively [17]. The most common phytotoxic compounds found in plants are terpenes and phenolic compounds [18, 19] that may inhibit or reduce germination capacity, may cause a delay in germination time and may hamper root elongation or nutrient absorption thereby reducing plant survival and growth [20–23]. The release of phytotoxic compounds may have ecological implications because non-competitive species can take advantage of the affected plants since the latter become less competitive [24]. Therefore, chemically-mediated interference might be a way for nurse plants to gain a competitive advantage over neighbouring plants that may be potentially competitive [17].

In arid and semi-arid areas of the Mediterranean region, chemical interference is likely to be a common phenomenon, given the abundance of aromatic plants that produce potential phytotoxic compounds [25–27]. This production may be promoted by stressful conditions as lack of water or nutrients, salinization and high solar radiation [28]. Furthermore, in arid and semi-arid environments, the effect of those compounds may increase because of their relative accumulation in the soil [29] and an intensification of plant sensitivity [28]. The accumulation of phytotoxic compounds in the soil may have incremental effects along plant life-span [29], being the longer-lived plants more affected by phytotoxic effects due to more prolonged exposure.

Mediterranean gypsum plant communities are mainly composed of well-adapted gypsophytes (i.e., gypsum soil specialists). However, in those communities the gypsovag (i.e., non-specialist) rosemary-leaved rockrose *Cistus clusii* Dunal often forms locally dominant populations that are associated with species-poor plant communities. *Cistus clusii*, a multi-branched perennial shrub (0.5–1.0 m tall), is distributed throughout the western Mediterranean region on alkaline soils, including gypsum, marls and limestones [30], and is very tolerant to dry environments [31]. This shrub may have nurse potential because it provides shade under its canopy due to its multi-branched architecture, creating favourable microenvironments in which other species can establish [32]. Nevertheless, *C. clusii* commonly grows in patches isolated from other species (personal observation) which suggests that it may exert interference on other plant species in the community.

Several studies have shown that other *Cistus* species have phytotoxic effects, either inhibiting the germination and growth of hetero-specific seedlings via foliar exudates (*e.g., Cistus ladanifer*; [33–35]) or immobilising nutrients in the soil via root exudates (*e.g.*, *Cistus albidus*; [36]). Based on the species-poor patches of *C. clusii* observed in gypsum plant communities and the phytotoxicity of other *Cistus* species, we postulated that the gypsovag *C. clusii* could exert phytotoxic effects on other plants beneath its canopy. To date, no study has demonstrated phytotoxicity in *C. clusii*, even though it is known to produce phenolic compounds and terpenes [37–39].

The aim of this study was to test whether or not *C. clusii* interferes with neighbouring species in gypsum plant communities through chemical mechanisms of interference. A controlled seeding experiment was performed in a greenhouse to identify potential phytotoxic effects of aqueous extracts from *C. clusii* leaves, roots and a mixture of both plant tissues on the development of neighbouring species in the community. We hypothesized that (1) aqueous extracts from *C. clusii* would affect those species negatively and more specifically, we expected that (2) the effect of leaf extracts would be more intense than the effect of root extracts, as previously shown for other species [40]. Moreover, we expected the effect of the mixture of leaf and root extracts to be the most significant through a combined activity of both extracts. We predicted that (3) affections by aqueous extracts from *C. clusii* would be exhibited on seed germination inhibition or delay, early survival decline and plant growth reduction [20–23]. Complementary to the greenhouse experiment, plant spatial associations were evaluated in the field in the local vicinity of *C. clusii*, compared to a shrub species of similar architecture in the community. Plant spatial associations were assessed as an indicator for biotic interactions to disentangle the potential interference exerted by this shrub on neighbouring plants in gypsum plant communities. We hypothesized that (4) interference exerted by *C. clusii* would imply less plant–plant associations, resulting in an impoverishment of species in its local vicinity compared to the other shrub. Since the effects of phytotoxic compounds can increase along plant life-span [29], we expected 5) a more evident depletion of perennial plants (especially at adult stage) than of annual plants in the local vicinity of *C. clusii*.

## Methods

### Study area

The study was conducted in Sierra de Alcubierre (41°41′N 0°32′W, municipality of Leciñena), in the Middle Ebro Valley, Zaragoza (NE Spain), one of the largest gypsum outcrops in Europe [41]. This area has a semi-arid Mediterranean climate with high continental influence. Average precipitation is 367 mm year^−1,^ and average annual temperature is 14.5 °C (Zuera ‘Aspasa’ meteorological station, 1973–2012 period; source: Gobierno de Aragón, http://opendata.aragon.es). The landscape is characterised by low hills (480 m a.s.l. average) with mainly gypsiferous lithology, and flat valleys, most of which have been cultivated. In the gypsiferous hills, plant communities are composed predominantly of highly specialised flora (the gypsophytes *Helianthemum squamatum* (L.) Pers., *Gypsophila struthium* Loefl. ssp. *hispanica* (Willk.) G. López, *Ononis tridentata* L. and *Lepidium subulatum* L.) and some widespread Mediterranean shrub species, *e.g., Rosmarinus officinalis* L., *Thymus vulgaris* L. and *C. clusii* [42]. The vegetation structure is a scattered scrubland comprising large open areas interspersed with patches of vegetation. This unique habitat (*Gypsophiletalia*) has a high ecological value and is listed as a conservation priority in international directives [43].

### Greenhouse experiment

A greenhouse seeding experiment was performed under controlled conditions to identify potential phytotoxic effects of *C. clusii* leaf and root aqueous extracts on the germination, early survival and growth of nine species. The choice of species included the most abundant perennials co-occurring with *C. clusii* at the study area [44]. Selected species were *G. struthium* ssp. *hispanica* (hereafter *G. struthium*), *H. squamatum*, *Helianthemum syriacum* (Jacq.) Dum. Cours., *R. officinalis*, *T. vulgaris*, *Helichrysum stoechas* (L.) Moench, *Linum suffruticosum* Orteg. ex Planch. and S*tipa lagascae* Roem. & Schult. *Cistus clusii* was also included in the experiment to test autotoxicity. Ripe fruits were collected at the study site from ten similar-sized individuals per test species. Most species seeds were collected in June 2015, except for *G. struthium* seeds, which were collected in September 2014 and *R. officinalis* seeds, which were collected in February 2015, matching the fructification peak respectively. Seeds were separated from the fruits, discarding any malformed seeds.

Aqueous solutions were prepared from leaves and roots of *C. clusii* and used as watering treatments in the experiment. Water was used as a solvent to simulate the leaching of phytotoxic compounds by rainfall. Solutions were prepared by ‘cool extraction’, soaking fresh plant material in distilled water for 24 h at room temperature in darkness [45]. For fresh material, we used leaves recently collected from natural communities and roots from plants grown for 3 months in a nursery. It was unfeasible to collect a sufficient amount of roots from natural communities given the difficulties encountered due to the deep taproots of *C. clusii* [46]. The seeds used to grow *C. clusii* in the nursery were collected from the same population as the collected leaves. The treatments were aqueous leaf extracts 1.5 g l^−1^ concentration (L), aqueous root extracts 0.025 g l^−1^ concentration (R), a mixture of both extracts 1.525 g l^−1^ concentration (RL) and water as the control (C). The water: leaf and water: root volumetric ratios were equivalent, and were within the range that occurs in natural conditions (see Additional file 1).

The seeding experiment was performed in July 2015 in a greenhouse maintained at 25 °C during the day and 15 °C during the night. Trays (60 × 40 × 20 cm) were filled with a peat-based substrate in which a known seed mixture was sown (9 species × 15 seeds per species in each tray). Seeding density was 0.06 seeds cm^−2^. Each of the four extract treatments (L, R, RL and C) had five replicates (trays). To assure germination, hard seeds were pretreated to break coat-imposed dormancy [47]; specifically, *H. squamatum*, *H. syriacum* and *T. vulgaris* seeds were mechanically scarified using sandpaper [48] and *C. clusii* seeds received a dry-heat shock at 100 °C for 5 min [49]. Before sowing, all seeds were soaked in distilled water for 20 h to stimulate germination. Extract treatments (L, R, RL and C) were applied twice a week by watering trays with one litre of the specific aqueous solution. To record potential effects of the extract treatments on the delay of germination and possible cumulative effects on seedling survival, the experiment was monitored once per week. Germinated seedlings were labeled with a toothpick indicating the date of emergence, and seedling survival was recorded throughout the experiment. To avoid any position effects, trays were randomized once a week. After 10 weeks, living seedlings were harvested and washed, and the below-ground and above-ground parts of each plant were separated and kept in individual paper bags. Plants were dried in an oven at 70 °C for 48 h and weighed using a 0.01 mg precision balance. Total dry biomass and the ratio of below-ground/above-ground biomass were used as growth estimators.

### Vegetation survey

To test the potential interference exerted by *C. clusii* on neighbouring plants under natural conditions, a vegetation survey was conducted in May 2014, at the peak of vegetation growth. We surveyed the plants growing under the canopy of *C. clusii*, and also under the canopy of *G. struthium* for comparison purpose. The latter is a gypsophyte shrub which has a proved nurse role [50] and, to our knowledge, without any phytotoxic effects. Both shrubs have similar architecture, providing similar soil temperature and surface compaction under their canopies, which were improved, compared to open patches (see Additional file 2). To obtain comparable samples from the surrounding open patches, we surveyed the vegetation in paired areas placed in a random direction ≥ 50 cm away from each sampled target plant. Sampled areas were defined by circles matching the size of the area under the canopy of the paired target plant [51]. For both focal species, 25 sets of paired plant-open patches were sampled (n = 100 circles). All plants growing within the circles were recorded and identified to the species level. To assess the potentially differential phytotoxic effects along plant life-span, plants were categorised as either annual (short-lived) or perennial (long-lived), and within perennial, as either seedling or adult. For each category, we estimated richness (number of species present) and abundance (number of individuals present of all plant species) at each microsite: in open patches, under the canopy of *C. clusii*, and under the canopy of *G. struthium*.

### Data analyses

In the greenhouse experiment, the effects of the extract treatments either on total germination, as well as germination delay or on seedling survival gradual decline were evaluated considering germination and survival rates through time. Differences among extract treatments in seed germination rate and seedling survival rate of the nine test species were evaluated using Cox proportional hazard models and, for data visualisation, Kaplan–Meier curves [52]. Pairwise comparisons among extract treatments were performed with Tukey’s post hoc tests. For each test species, differences in total biomass and the ratio of below-ground/above-ground biomass among extract treatments were tested using linear mixed models (LMMs) with the tray as a random factor. Time since germination and size of seedlings were strongly correlated; therefore, the number of weeks from germination to the end of the experiment was included as a covariate. To attain the assumption of normality, the dependent variables were log-transformed.

Differences in richness and abundance among microsites were analysed by fitting generalized linear models (GLMs) with the assumption of a Poisson error distribution and log link function. The size of the sampled area (circle area) was included as a continuous covariate because it might have influenced the number of plants recorded. When a significant effect of the microsite was found, Tukey’s post hoc tests were applied for pairwise comparisons. In addition, for each of the nine test species used in the greenhouse experiment, G-tests (log likelihood ratio tests) were implemented to compare the observed frequencies with the haphazardly expected frequencies at each microsite (in open patches, under the canopy of *C. clusii*, and under the canopy of *G. struthium*). The expected frequencies were estimated as the total observed frequency of each species multiplied by the proportion of the area occupied by each microsite.

All statistical analyses were performed using R software [53]. To fit Cox models and construct Kaplan–Meier curves for germination and survival, the ‘survival’ package was used [54]. To fit LMMs for growth, the ‘nlme’ package was used [55]. To fit GLMs for richness and abundance, the ‘stats’ package was used [53]. All pairwise comparisons were performed using the ‘multcomp’ package [56].

## Results

### Greenhouse experiment

**Fig. 1:**
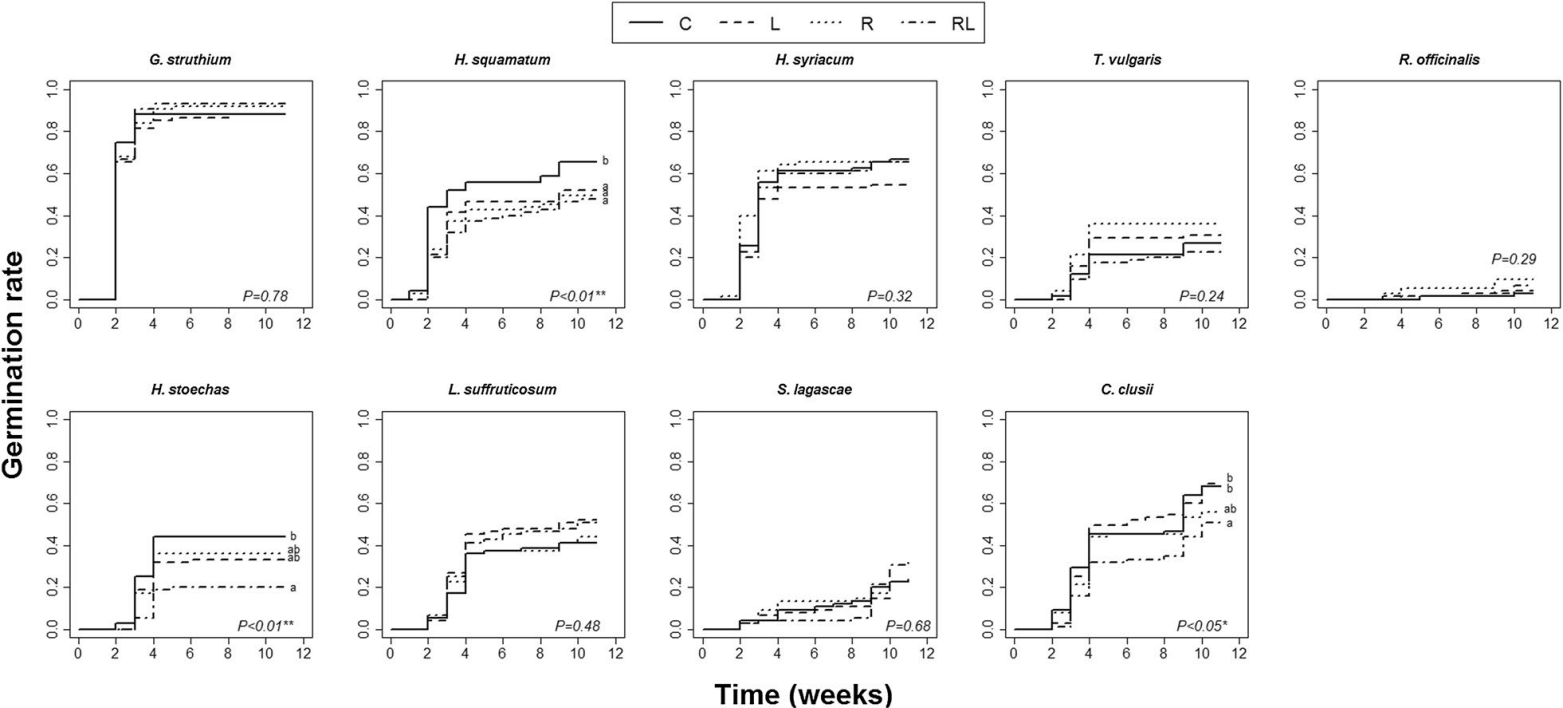
Kaplan-Meier curves representing seed germination rate over time (weeks from the experiment start) under each extract treatment: C (control), L (leaf extracts), R (root extracts) and RL (root and leaf extracts mixture) for each test species separately. Different letters represent statistically significant differences between extract treatments after Tukey’s post hoc tests (*P *< 0.05)

*Cistus clusii* aqueous extracts had a negative effect on seed germination rates of three of the nine test species (*H. squamatum*, *H. stoechas* and *C. clusii*; Fig. 1). Seed germination of *H. squamatum* was lower in the trays subjected to extracts than it was in the control trays and did not differ significantly among extract treatments. Seed germination of *H. stoechas* was lower in all extract treatments compared to the control treatment, being the lowest in the RL treatment. Seed germination of *C. clusii* was significantly lower in the R and the RL treatments than it was in the control treatment; however, germination rates did not differ significantly between the trays watered with leaf extracts (L) and the control trays. At the end of the experiment, < 10% of *R. officinalis* seeds had germinated (Fig. 1); therefore, this species was excluded from the survival and growth analyses.

**Fig. 2:**
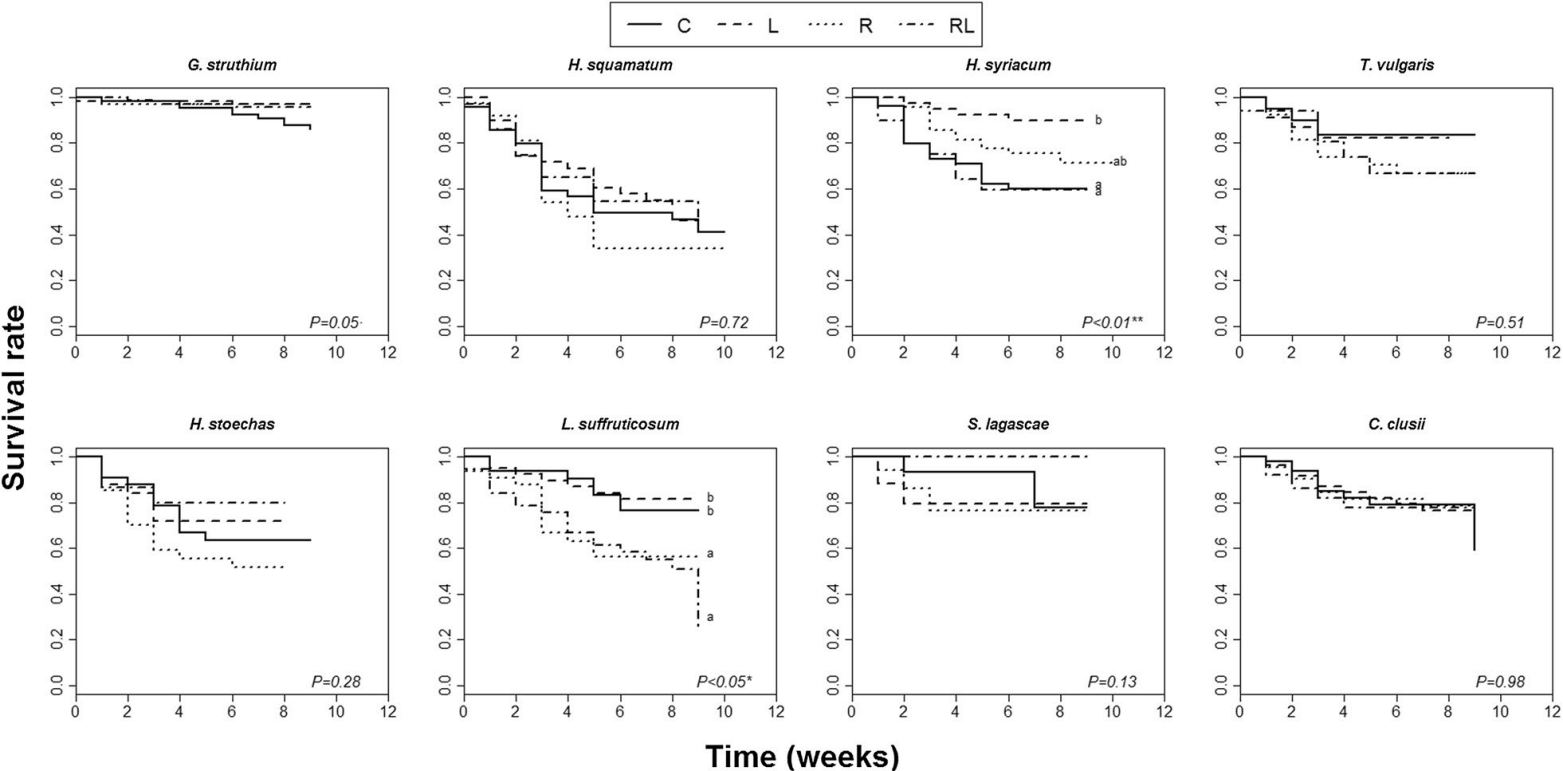
Kaplan-Meier curves representing seedling survival rate over time (weeks since germination) under each extract treatment: C (control), L (leaf extracts), R (root extracts) and RL (root and leaf extracts mixture) for each test species separately. Different letters represent statistically significant differences between extract treatments after Tukey’s post hoc tests (*P *< 0.05)

Survival rates differed significantly among extract treatments in two of the test species (*H. syriacum* and *L. suffruticosum*). Seedling survival of *L. suffruticosum* was lower in the R and the RL treatments compared to both the L treatment and the control treatment. Seedling survival of *H. syriacum* was the lowest in the control treatment and the RL treatment and the highest in the L treatment (Fig. 2).

Neither total biomass nor the ratio of below-ground/above-ground biomass differed significantly among extract treatments for any of the test species (see Additional files 3, 4).

### Vegetation survey

**Fig. 3:**
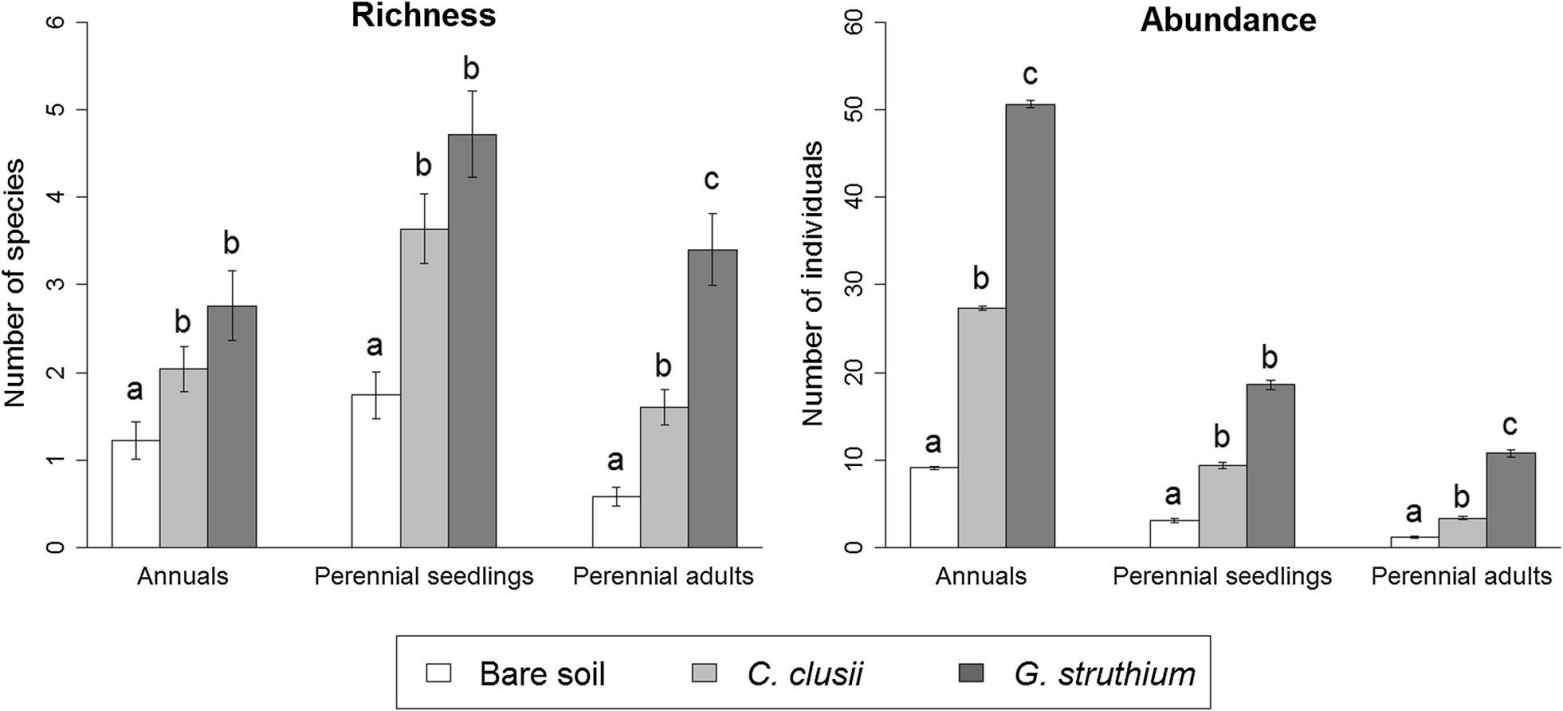
Mean richness and abundance of annuals, perennial seedlings and perennial adults among microsites: in open patches, under the canopy of *C. clusii* and under the canopy of *G. struthium*. Microsite effect was significant in GLMs in all cases (*P *< 0.001). Different letters indicate statistically significant differences between microsites after Tukey’s post hoc tests (*P *< 0.05)

Microsite had a significant effect on richness and abundance of annual and perennial plants (Fig. 3; see Additional file 5). In all cases, richness and abundance were lower in open patches than under the canopies of both shrubs. Significantly fewer species of perennial adults were found under the canopy of *C. clusii* than under the canopy of *G. struthium*; however, richness under the shrubs did not differ significantly for seedlings of perennial species and annual plants. The abundances of annual plants and perennial adult plants were lower under the canopy of *C. clusii* than under the canopy of *G. struthium*; however, the abundance of perennial seedlings did not differ significantly between the two shrubs (Fig. 3; see Additional file 5).

Except for *G. struthium*, *H. stoechas* and *S. lagascae*, the seedlings of the test species were significantly less frequent in open patches than they were under the canopy of *C. clusii*. Among adults, *H. syriacum* and *H. squamatum* were less frequent in open patches than they were under the canopy of *C. clusii*. The other test species showed no difference in frequency between these two microsites (Table 1; see Additional file 6). Most test species were significantly less frequent in open patches than they were under the canopy of *G. struthium,* except seedlings of *S. lagascae* and adults of *H. squamatum* and *R. officinalis* (Table 1; see Additional file 6). Five of the test species were significantly less frequent under the canopy of *C. clusii* than they were under the canopy of *G. struthium*; specifically, seedlings of *H. syriacum*, *H. stoechas* and *C. clusii*, and adults of *T. vulgaris*, *H. stoechas*, *S. lagascae* and *C. clusii*. The observed frequency of *C. clusii* seedlings under the canopy of *C. clusii* was significantly higher than expected (Table 1; see Additional file 6).

**Table 1:** Summary of the pairwise comparisons of the G-test, indicating significant differences (P < 0.05) between observed frequencies (f) and expected frequencies (f_e_) of the nine test species at each microsite

	f (f_e_)_Op_	f (f_e_)_Cc_	G	*P*	f (f_e_)_Op_	f (f_e_)_Gs_	G	*P*	f (f_e_)_Cc_	f (f_e_)_Gs_	G	*P*
Seedlings												
*G. struthium*	8 (11)	6 (3)	2.51	0.057	8 (14)	14 (8)	5.83	*< 0.01*	6 (7)	14 (13)	0.11	0.372
*H. squamatum*	2 (21)	26 (7)	61.71	*< 0.001*	2 (29)	45 (18)	72.14	*< 0.001*	26 (24)	45 (47)	0.33	0.284
*H. syriacum*	6 (10)	7 (3)	74.98	*< 0.001*	6 (25)	35 (16)	202.94	*< 0.001*	7 (14)	35 (28)	147.02	*< 0.001*
*T. vulgaris*	43 (76)	56 (23)	49.27	*< 0.001*	43 (90)	103 (56)	62.88	*< 0.001*	56 (53)	103 (106)	0.23	0.314
*R. officinalis*	18 (24)	14 (8)	6.25	*< 0.01*	18 (27)	25 (16)	7.02	*< 0.01*	14 (13)	25 (26)	0.11	0.371
*H. stoechas*	6 (7)	3 (2)	0.43	0.255	6 (33)	48 (21)	60.63	*< 0.001*	3 (17)	48 (34)	22.79	*< 0.001*
*L. suffruticosum*	21 (34)	23 (10)	16.79	*< 0.001*	21 (32)	30 (19)	8.90	*< 0.01*	23 (18)	30 (35)	2.28	0.065
*S. lagascae*	5 (6)	3 (2)	0.77	0.190	5 (6)	5 (4)	0.58	0.223	3 (3)	5 (5)	0.06	0.404
* C. clusii*	21 (50)	44 (15)	56.52	*< 0.001*	21 (46)	54 (29)	35.34	*< 0.001*	44 (33)	54 (65)	5.56	*< 0.01*
Adults												
*G. struthium*	0 (−)	0 (−)	–	–	0 (–)	0 (–)	–	–	0 (–)	0 (–)	–	–
*H. squamatum*	5 (8)	5 (2)	3.26	*< 0.05*	5 (7)	6 (4)	1.21	0.135	5 (4)	6 (7)	0.69	0.204
*H. syriacum*	3 (6)	5 (2)	5.46	*< 0.05*	3 (15)	21 (9)	25.28	*< 0.001*	5 (9)	21 (17)	2.58	0.054
*T. vulgaris*	12 (12)	4 (4)	0.02	0.449	12 (38)	49 (23)	45.50	*< 0.001*	4 (18)	49 (35)	20.25	*< 0.001*
*R. officinalis*	3 (3)	1 (1)	0.00	0.474	3 (2)	1 (2)	0.31	0.289	1 (1)	1 (1)	0.23	0.314
*H. stoechas*	2 (2)	0 (0)	1.08	0.150	2 (6)	8 (4)	7.34	*< 0.01*	0 (3)	8 (5)	6.50	*< 0.01*
*L. suffruticosum*	0 (−)	0 (−)	–	–	0 (2)	3 (1)	5.78	*< 0.01*	0 (1)	3 (2)	2.44	0.059
*S. lagascae*	0 (−)	0 (−)	–	–	0 (6)	10 (4)	19.28	*< 0.001*	0 (3)	10 (7)	8.13	*< 0.01*
*C. clusii*	7 (6)	1 (2)	0.63	0.214	7 (11)	11 (7)	3.87	*< 0.05*	1 (4)	11 (8)	4.25	*< 0.05*

*Op* open patches, *Cc* under the canopy of *C. clusii*, *Gs* under the canopy of *G. struthium*

## Discussion

We combined a controlled experiment with a field survey aiming to disentangle the potential chemical mechanisms of interference exerted by *C. clusii* on neighbouring plant species in gypsum plant communities of the Middle Ebro Valley. While the controlled experiment allowed us to isolate the phytotoxic effect of *C. clusii* root and leaf aqueous extracts on the early establishment of the test species, the field survey showed a more complex picture. In the field, chemical interference influences the net plant–plant interactions outcome together with facilitation and competition for resources [57]. Thus, a complementary assessment considering experimental and field effects of *C. clusii* on our test species can help unravel the relative relevance of its potential chemical interference compared to other types of interference, i.e., competition for resources (Table 2).

**Table 2:** Comparison of the potential effects exerted by *C. clusii* under experimental and field conditions

	Experimental conditions		
	Negative effect	No effect	Positive effect
Field conditions			
Negative effect	Chemical interference: *H. stoechas, C. clusii* (adults)	Other sources of interference (i.e., competition for resources), and phytochemicals accumulation and/or transformation in soils: *T. vulgaris*, *S. lagascae*	Other sources of interference (i.e., competition for resources), and phytochemicals accumulation and/or transformation in soils: *H. syriacum*
No effect	Neutral interaction outcome (facilitation + chemical interference): *H. squamatum*, *L. suffruticosum*	*C. clusii*-tolerant species: *G. struthium*, *R. officinalis*	–
Positive effect	Limited seed dispersal: *C. clusii* (seedlings)	–	–

The greenhouse experiment confirmed our hypothesis that aqueous extracts of *C. clusii* affect the development of some species from gypsum plant communities. Supplementary chemical analyses of *C. clusii* tissues confirmed the presence of water-soluble terpenes and phenolic compounds with potential phytotoxic activity (see Additional file 7). We hypothesised that aqueous extracts from *C. clusii* would affect germination rates, survival rates and growth of the test species negatively. Even though it was not visible for all test species, our hypothesis was supported by experimental results for germination and survival rates. However, it was not evident for seedling biomass, manifesting that *C. clusii* aqueous extracts do not affect seedling growth, at least at the short-term. Chemically inhibition or retardation of germination and seedling survival decline may have ecological implications in the community. This may result in an advantage of low-competitive species over the affected species at early life stages [21, 24], likely causing a species shift in the community. Germination inhibition by phytotoxic compounds is a phenomenon widely reported by other studies in semi-arid communities [21, 58].

Diverse effects of aqueous extracts were found on germination and survival rates depending on the plant tissue tested in the experiment. Other studies found that leaves of allelopathic plants contained more water-soluble phytotoxic compounds than roots [59], likely resulting in stronger phytotoxic effects, as observed by Dorning and Cipollini [40] in an invasive shrub. Our additional chemical analyses confirmed that leaves from *C. clusii* contain more water-soluble potential phytotoxic compounds than its roots. We expected the leaf extracts to exert a stronger negative effect on the test species than root extracts. On the contrary, despite containing fewer compounds than did its leaves, solutions containing root extracts more often had negative effects on germination and seedling survival compared to pure leaf extracts. As predicted, roots combined with leaves was the most inhibiting treatment likely due to a synergic effect of the compounds contained in both plant tissues [60].

The outcomes of the field survey and the experiment denoted that chemical interference could explain why some species are less frequent in the local vicinity of *C. clusii*. This fact was especially evident for *H. stoechas* because the low number of individuals found under the canopy of *C. clusii* compared to those under the canopy of *G. struthium* paralleled the low germination of seeds treated with *C. clusii* extracts. These results confirm our hypothesis of the lessening of plant–plant associations due to the chemical interference exerted by *C. clusii* on neighbouring plants, deriving to an impoverishment of species around this shrub. It has already been evidenced in semi-arid plant communities that phytotoxic effects on neighbouring plants result in species-poor islands around the allelopathic plant [61]. Other negative interactions between *C. clusii* and neighbouring species in the field could not be corroborated in our seeding experiment because other factors beyond the releasing of phytotoxic compounds may influence plant establishment under natural conditions [62]. For example, in the field, fewer than the expected number of individuals of *H. syriacum* were found under the canopy of *C. clusii*; however, in the experiment, *C. clusii* aqueous extracts had a positive effect on *H. syriacum* seedling survival. Similarly, *T. vulgaris* and *S. lagascae* were found in low abundance under the canopy of *C. clusii*; however, the extract treatments did not significantly affect their performance. Negative interactions between those species and *C. clusii* are thus more likely caused by competition for space and resources rather than chemical interference [57].

The limitations of the greenhouse experiment could have led to an underestimation of the phytotoxic effects of *C. clusii* that may occur under natural conditions. For example, the effects of hydrophobic compounds present in *C. clusii* (*e.g.,* β-pinene; [39]) were not tested in the experiment because they were not extracted in the aqueous solutions (see Additional file 4). Those compounds can be released to the environment by volatilisation and are potentially phytotoxic [58, 63]. Also, soil microorganisms are known to transform chemical compounds [64], which can increase phytotoxicity under natural conditions [65], dissimilar to the controlled conditions of the experiment. On the other hand, it should be noted that phytotoxic compounds can have a cumulative effect over a plant life-span [29] and our seeding experiment did not last long enough to detect potential long-term negative effects of *C. clusii*. Nevertheless, accordingly to our expectations, long-term negative effects were found in the field survey since *C. clusii* consistently harboured fewer species than *G. struthium*, mainly perennials at adult stages.

Both in the greenhouse experiment and in the field *C. clusii* did not show a negative effect on *G. struthium*, suggesting that this species tolerates *C. clusii*. In the field, *R. officinalis* also appeared to exhibit tolerance to *C. clusii*; however, this could not be confirmed experimentally because *R. officinalis* exhibited an overall very low germination rate. Those species may have adapted to the potential phytotoxic compounds of *C. clusii* because they frequently co-occur with this species in gypsum plant communities of the Middle Ebro Valley. Tolerance to ‘chemical neighbours’ is a well-studied co-evolutionary phenomenon that allows a species to coexist with phytotoxic plant species [61, 66, 67]. Moreover, although *C. clusii* extracts reduced seedling survival of *H. squamatum* and *L. suffruticosum* in the greenhouse experiment, this was not evident in natural conditions. Possibly, in nature, the net interaction outcome between *C. clusii* and those species tends to be neutral because of positive interactions. *Cistus clusii* has the potential of behaving as a nurse plant since the area under its canopy provides microsites similar to known nurse plants in this habitat (*e.g., G. struthium;* [50]) and may suit the establishment of *C. clusii*-tolerant species [4]. Indeed, there was a positive net effect of *C. clusii* when richness and plant abundance were compared to open patches. Positive effects of *C. clusii* on the establishment of other species compared to open patches had been documented in semi-arid plant communities before [32].

Few of the studies that investigated phytotoxicity in plants also evaluated autotoxicity [33]. In the greenhouse experiment, *C. clusii* aqueous extracts inhibited the germination of its own seeds, indicating a phytotoxic potential against itself. In natural conditions, more seedlings of *C. clusii* than expected were found under its canopy, probably because of high seed accumulation [68]; however, fewer than the expected numbers of adult *C. clusii* were found under its canopy, in agreement with an auto-inhibiting effect. The low establishment of adult *C. clusii* in the vicinity of *C. clusii* shrubs could have important implications for *C. clusii* population dynamics as auto-inhibition could lead to a strong reliance on other nurse species to establish under the highly restrictive conditions that occur in gypsum environments.

Despite ameliorating micro-environmental conditions under its canopy (see Additional file 2), and having a nurse role compared to open patches [32], *C. clusii* did not present such a positive role as the other shrub with similar architecture, suggesting interference with neighbouring plants in the community. We found potential phytotoxic compounds in *C. clusii* leaves and roots, and the associated vegetation showed a species-specific sensitivity to *C. clusii*. Among test species, there were possible *C. clusii*-vulnerable species (*H. stoechas*, *H. syriacum*, *S. lagascae*, and *T. vulgaris*), but also potential *C. clusii*-tolerant species (*G. struthium*, *H. squamatum*, *L. suffruticosum*, and *R. officinalis*). Species-specific phytotoxicity has been previously described [27] and might have important ecological implications for the dynamics of plant communities, by affecting the recruitment of some species and thereby their abundance in the community [69]. Besides *C. clusii*, other gypsovags that are very common in gypsum environments (*e.g., T. vulgaris* and *R. officinalis*) leach chemical compounds with known phytotoxic activity to their local environments [70, 71]. Phytotoxicity might be a mechanism that allows gypsovags to succeed in competition for resources with neighbouring plant species that may be better adapted to the harsh conditions in gypsum soils [72]. For example, phosphorous is scarce in gypsum hills [73, 74], and excluding other plants from the local vicinity might be a means of minimising local phosphorous depletion.

## Conclusions

This study provides novel results of species-specific interference of *C. clusii* on other plant species. Phytotoxicity of *C. clusii* at least partly affects species richness in its local vicinity in gypsum plant communities in the Middle Ebro Valley. The importance of the role of the phytotoxicity of *C. clusii* in plant–plant interaction outcomes at the community level should be investigated in other Mediterranean plant communities.

## Additional files

**Additional file 1.** Methods and results for the determination of aqueous extracts concentration.

**Additional file 2.** Methods and results for the micro-environmental conditions under the canopies of *C. clusii* and *G. struthium*.

**Additional file 3.** Effects of the extract treatments on seedling biomass.

**Additional file 4.** Dataset including the data about seed germination, seedling survival and seedling biomass from the greenhouse experiment.

**Additional file 5.** Dataset including the data about richness and abundance from the field surveys.

**Additional file 6.** Dataset including the data about the observed and expected frequencies of the test species under field conditions.

**Additional file 7.** Methods and results for the chemical analyses of leaf and root extracts.

## Abbreviations

L: aqueous leaf extract
R: aqueous root extract
RL: aqueous root and leaf extract
C: control (water)
LMMs: linear mixed models
GLMs: generalized linear models
