# Supplementary material for: Species-specific interference exerted by the shrub Cistus clusii Dunal in a semi-arid Mediterranean gypsum plant community
Source: BMC Ecol. 2018 Nov 29;18:49. doi: 10.1186/s12898-018-0204-x (PMC6267893; doi:10.1186/s12898-018-0204-x)
Supplement: Supplementary file 1 — Additional file 1. Methods and results for the determination of aqueous extracts concentration. [file 12898_2018_204_MOESM1_ESM.pdf]

Additional file 1. Determination of extracts concentration

Realistic concentrations of extracts were determined considering the volume of water from rainfall that would pass through a surface area of live material equivalent to the area of a tray used in the greenhouse experiment (Orr et al. 2005). Water volume received in that surface (60 cm x 40 cm = 2400 cm^2) was calculated considering average annual rainfall in the study site (367 $mm \cdot year^{-1}$; Zuera ‘Aspasa’ meteorological station, 1973-2012 period; source: Gobierno de Aragón, http://opendata.aragon.es). Then, fresh leaves covering 2400 cm^2 of soil surface in the field were collected from five individuals of *C. clusii* and consequently fresh biomass was weighed using a 0.01 g precision balance. Thus, leaf extract concentration was determined using mean biomass of leaves per individual (81.19 g; Table S1) and mean water volume passing through a surface of 2400 cm^2 in the field (88.08 l). The resulting mass concentration of leaf extracts under natural conditions was approximately 1 $g \cdot l^{-1}$. We formulated root extracts to the same volumetric ratio as leaf extracts, considering that below-ground material occupies the same surface than above-ground material in *C. clusii* (Guerrero-Campo 1998). Roots were lighter than leaves, therefore, the resulting mass concentration in root extracts was lower than in leaf extracts.

Table S1. Fresh weight of leaves collected from five individuals of *C. clusii* covering in natural communities.

**Individual**	**Plant height (cm)**	**Leaves biomass (g)**
Cistus 1	54	48.34
Cistus 2	72	99.83
Cistus 3	60	91.44
Cistus 4	45	62.67
Cistus 5	43	103.66
**Mean**	54.8	81.19

References

Guerrero-Campo J (1998) *Respuestas de la vegetación y de la morfología de las plantas a la erosión del suelo: Valle del Ebro y Prepirineo aragonés*. Consejo de Protección de la Naturaleza de Aragón.

Orr SP, Rudgers JA, Clay K (2005) Invasive plants can inhibit native tree seedlings: Testing Potential Allelopathic Mechanisms. *Plant Ecology* **181**:153–165
