# Supplementary material for: Species-specific interference exerted by the shrub Cistus clusii Dunal in a semi-arid Mediterranean gypsum plant community
Source: BMC Ecol. 2018 Nov 29;18:49. doi: 10.1186/s12898-018-0204-x (PMC6267893; doi:10.1186/s12898-018-0204-x)
Supplement: Supplementary file 2 — Additional file 2. Methods and results for the micro-environmental conditions under the canopies of C. clusii and G. struthium. [file 12898_2018_204_MOESM2_ESM.pdf]

Additional file 2. Amelioration of micro-environmental conditions under the canopies of *Cistus clusii* and *Gypsophila struthium* ssp. *hispanica*

We measured soil compaction, soil humidity, and soil temperature under the canopy of 15 random individuals of *C. clusii*, 15 random individuals of *G. struthium* and at 15 random points in open patches (n=45) in spring. Soil compaction was measured as surface mechanical resistance (kg·cm^{-2}) with a force gauge equipped with a compression plate with a diameter of 2 cm (MECMESIN Basic Force Gauge 500N). Soil humidity (6 cm maximum depth) was measured as the soil volumetric water content (%) with a ML3 ThetaProbe soil moisture sensor (Delta-T Devices). Finally, surface soil temperature (6 cm deep) was measured with a T-bar digital stem thermometer (ATM Ltd ST-9265A). In order to check the influence of plant size on soil physical properties, volume of all individuals was measured.

In addition, we collected soil samples under the canopy of five random individuals of *C. clusii*, five random individuals of *G. struthium* and at five random points in open patches (n=15). Soil samples were dried and sieved over a 2 mm mesh sieve before the analyses. We analyzed available phosphorous (P), total organic carbon (TOC), total carbon (C) and total nitrogen (N) in all soil samples. Available phosphorous of samples extracted with Bray nº1 reagent (Bray and Kurtz 1945) was estimated using a spectrometer (UNICAM 8625 UV/Vis Spectrometer) with the absorbance at 430 nm. To analyze total organic carbon, samples were first submitted to a chromatic acid digestion (Heanes 1984), and then this was estimated using a spectrometer (UNICAM 8625 UV/Vis Spectrometer) with the absorbance at 590 nm. Finally, soil samples were

ground to a fine powder and then total carbon and total nitrogen were measured in a Vario MAX CN analyzer (Elementar Vario MAX CN).

To test the effect of the microsite on physical-chemical soil properties, ANCOVAs were performed considering plant volume as a covariate. Pairwise comparisons among target species were performed with Bonferroni correction for all the soil properties analyzed. Statistical analyses were performed in R (R Development Core Team 2014).

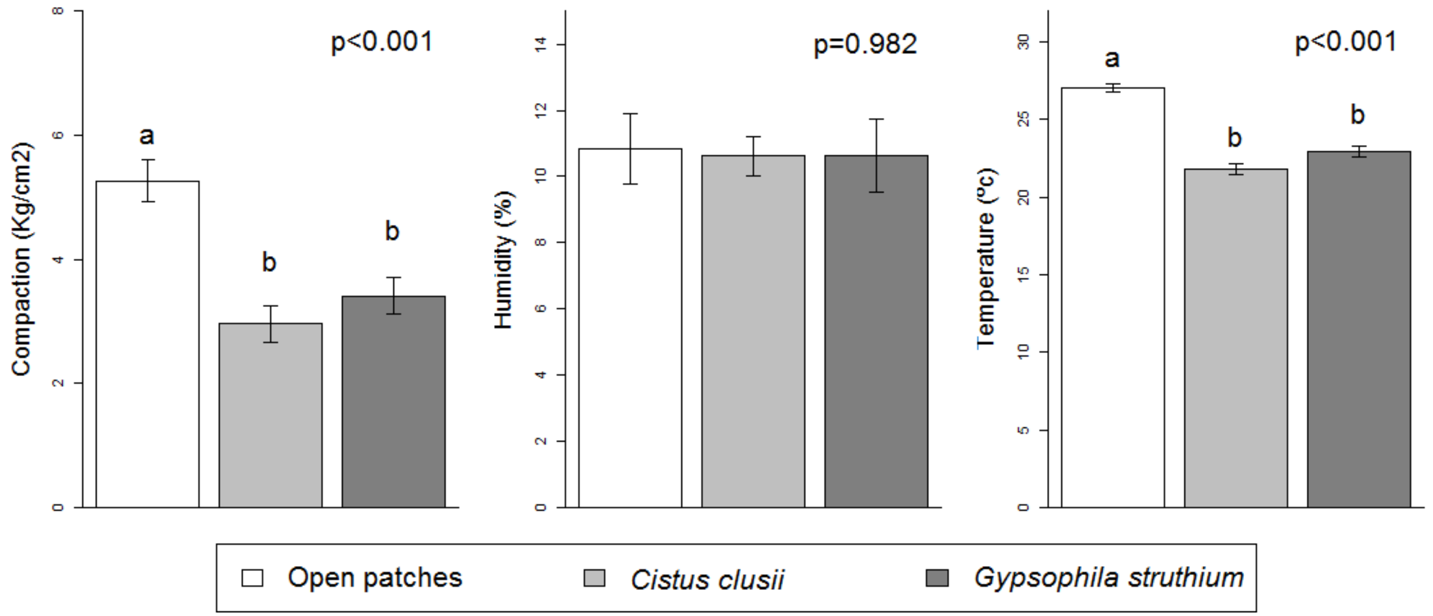

Figure S1. Mean soil compaction (Kg·cm^{-2}), soil humidity (% volumetric water content) and soil temperature (ºC) measured in different microsites: in open patches, under the canopy of *C. clusii* and under the canopy of *G. struthium*. The microsite effect was significant when p<0.05 in ANOVAs. Different letters indicate statistically significant differences among microsites after pairwise comparisons with Bonferroni correction.

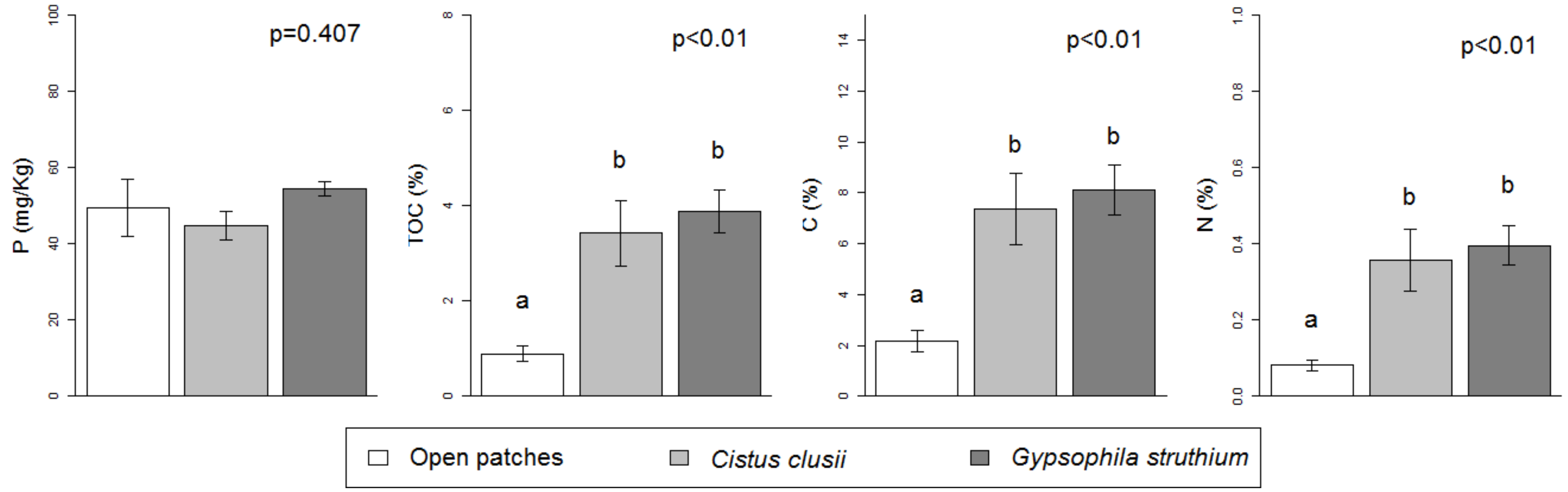

Figure S2. Mean values of available phosphorous (P), total organic carbon (TOC), total carbon (C) and total nitrogen (N) of soil samples collected from each microsite: in open patches, under the canopy of *C. clusii* and under the canopy of *G. struthium*. The microsite effect was significant when $p<0.05$ in ANOVAs. Different letters indicate statistically significant differences among target species after pairwise comparisons with Bonferroni correction.

References

Bray RH, Kurtz LT (1945) Determination of total, organic, and available forms of phosphorus in soils. *Soil Science* **59**:39–46

Heanes DL (1984) Determination of total organic-C in soils by an improved chromic acid digestion and spectrophotometric procedure. *Communications in Soil Science and Plant Analysis* **15**:1191–1213

R Development Core Team (2014) R: A language and environment for statistical computing. ISBN 3-900051-07-0, Vienna, Austria
