# Supplementary material for: Species-specific interference exerted by the shrub Cistus clusii Dunal in a semi-arid Mediterranean gypsum plant community
Source: BMC Ecol. 2018 Nov 29;18:49. doi: 10.1186/s12898-018-0204-x (PMC6267893; doi:10.1186/s12898-018-0204-x)
Supplement: Supplementary file 3 — Additional file 3. Effects of the extract treatments on seedling biomass. [file 12898_2018_204_MOESM3_ESM.pdf]

Additional file 3. Effects of the extract treatments on seedling biomass

Table S2. Mean values ± SE per extract treatment (C, L, R, RL) of the total biomass of the seedlings of the test species in the greenhouse experiment and summary of the LMMs implemented to test the effects of the extract treatments, the time since the germination time and the interaction among these factors on total biomass.

	**Mean values ± SE**				**Treatments**			**Time since germination**			**Treatments x time since germination**		
**Test species**	**C**	**L**	**R**	**RL**	**DF**	**F**	**p-value**	**DF**	**F**	**p-value**	**DF**	**F**	**p-value**
G. struthium	73.27 ± 6.58	110.69 ± 10.26	135.53 ± 14.14	90.19 ± 10.77	3	1.65	0.217	1	82.58	**<0.001**	3	2.23	0.086
H. squamatum	4.51 ± 0.85	3.92 ± 0.59	9.91 ± 3.33	5.12 ± 1.31	3	1.65	0.219	1	110.20	**<0.001**	3	1.40	0.256
H. syriacum	4.87 ± 0.55	8.35 ± 0.91	8.96 ± 1.54	6.78 ± 1.01	3	0.88	0.471	1	43.67	**<0.001**	3	0.02	0.995
T. vulgaris	6.25 ± 1.86	16.04 ± 2.18	21.72 ± 5.14	17.11 ± 5.44	3	0.88	0.473	1	46.99	**<0.001**	3	1.44	0.245
H. stoechas	6.85 ± 1.24	15.22 ± 2.57	13.75 ± 4.41	10.86 ± 4.45	3	1.24	0.332	1	2.29	0.134	3	0.99	0.407
L. suffruticosum	3.94 ± 0.73	5.38 ± 0.63	7.97 ± 2.24	4.44 ± 0.62	3	1.07	0.389	1	41.77	**<0.001**	3	2.52	0.066
S. lagascae	13.15 ± 7.96	10.34 ± 4.41	10.72 ± 3.57	2.46 ± 0.21	3	0.64	0.601	1	172.65	**<0.001**	3	0.23	0.875
C. clusii	3.61 ± 0.67	5.01 ± 0.80	8.52 ± 1.99	4.24 ± 1.28	3	0.33	0.805	1	374.91	**<0.001**	3	1.39	0.248

C: control, L: leaf extract, R: root extract, RL: root and leaf extract

Table S3. Mean values ± SE per extract treatment (C, L, R, RL) of the below-ground/above-ground biomass ratio of the seedlings of the test species in the greenhouse experiment and summary of the LMMs implemented to test the effects of the extract treatments, the time since the germination time and the interaction among these factors on below-ground/above-ground biomass ratio.

	**Mean values ± SE**				**Treatments**			**Time since germination**			**Treatments x time since germination**		
**Test species**	**C**	**L**	**R**	**RL**	**DF**	**F**	**p-value**	**DF**	**F**	**p-value**	**DF**	**F**	**p-value**
G. struthium	0.19 ± 0.01	0.17 ± 0.01	0.19 ± 0.02	0.18 ± 0.01	3	0.41	0.752	1	1.87	0.173	3	1.91	0.130
H. squamatum	0.18 ± 0.02	0.20 ± 0.04	0.22 ±0.02	0.21 ± 0.02	3	1.65	0.219	1	110.20	**<0.001**	3	1.40	0.256
H. syriacum	0.28 ± 0.02	0.26 ± 0.02	0.24 ± 0.01	0.25 ± 0.02	3	0.88	0.470	1	43.67	**<0.001**	3	0.02	0.995
T. vulgaris	0.34 ± 0.04	0.27 ± 0.03	0.23 ± 0.02	0.27 ± 0.03	3	0.88	0.473	1	46.99	**<0.001**	3	1.44	0.245
H. stoechas	0.26 ± 0.02	0.23 ± 0.02	0.24 ± 0.02	0.25 ± 0.04	3	1.24	0.332	1	2.29	0.138	3	0.99	0.407
L. suffruticosum	0.36 ± 0.03	0.30 ± 0.02	0.31 ± 0.03	0.29 ± 0.04	3	1.07	0.389	1	41.76	**<0.001**	3	2.52	0.066
S. lagascae	0.48 ± 0.03	0.51 ± 0.03	0.43 ± 0.03	0.46 ± 0.03	3	0.64	0.601	1	172.65	**<0.001**	3	0.23	0.875
C. clusii	0.26 ± 0.02	0.23 ± 0.01	0.25 ± 0.02	0.26 ± 0.02	3	0.33	0.805	1	374.91	**<0.001**	3	1.39	0.248

C: control, L: leaf extract, R: root extract, RL: root and leaf extract
