# Supplementary material for: Species-specific interference exerted by the shrub Cistus clusii Dunal in a semi-arid Mediterranean gypsum plant community
Source: BMC Ecol. 2018 Nov 29;18:49. doi: 10.1186/s12898-018-0204-x (PMC6267893; doi:10.1186/s12898-018-0204-x)
Supplement: Supplementary file 7 — Additional file 7. Methods and results for the chemical analyses of leaf and root extracts. [file 12898_2018_204_MOESM7_ESM.pdf]

## Additional file 7. Chemical analyses of leaf and root extracts

Chemical compositions of *C. clusii* leaves and roots were analysed by gas chromatography coupled with mass spectrometry on a Shimadzu GCMS-QP2010 with auto-sampler. The extracts were prepared by individually soaking 0.003 g of chopped leaves and 0.003 g of chopped roots in 1 ml of methanol for 24 hours at room temperature in darkness. Methanol was used as polar solvent to extract water-soluble compounds (Sokmen et al. 1999), which might be present in the aqueous extracts used in the experiment.

The GC-MS was equipped with a Supelco Omegawax 320 capillary column (length 30 m, inner diameter 0.32 mm, film thickness of 0.25 µm; Sigma Aldrich, Stockholm, Sweden). Samples (1 µl) were injected in split mode with a split ratio of 10:1. Helium was used as a carrier gas with a total flow of 22.7 $ml \cdot min^{-1}$ and a column flow at 1.79 $ml \cdot min^{-1}$. The injection temperature was 220°C and the oven programmed as follows: initial temperature of 50 °C for 2 min and then increased to 240 °C with a rate of 20 $°C \cdot min^{-1}$. The mass spectrometer was operated in electron ionization mode at 70 eV. The ion source temperature was 250 °C. Individual compounds were identified by comparing the obtained mass spectra results to known standards.

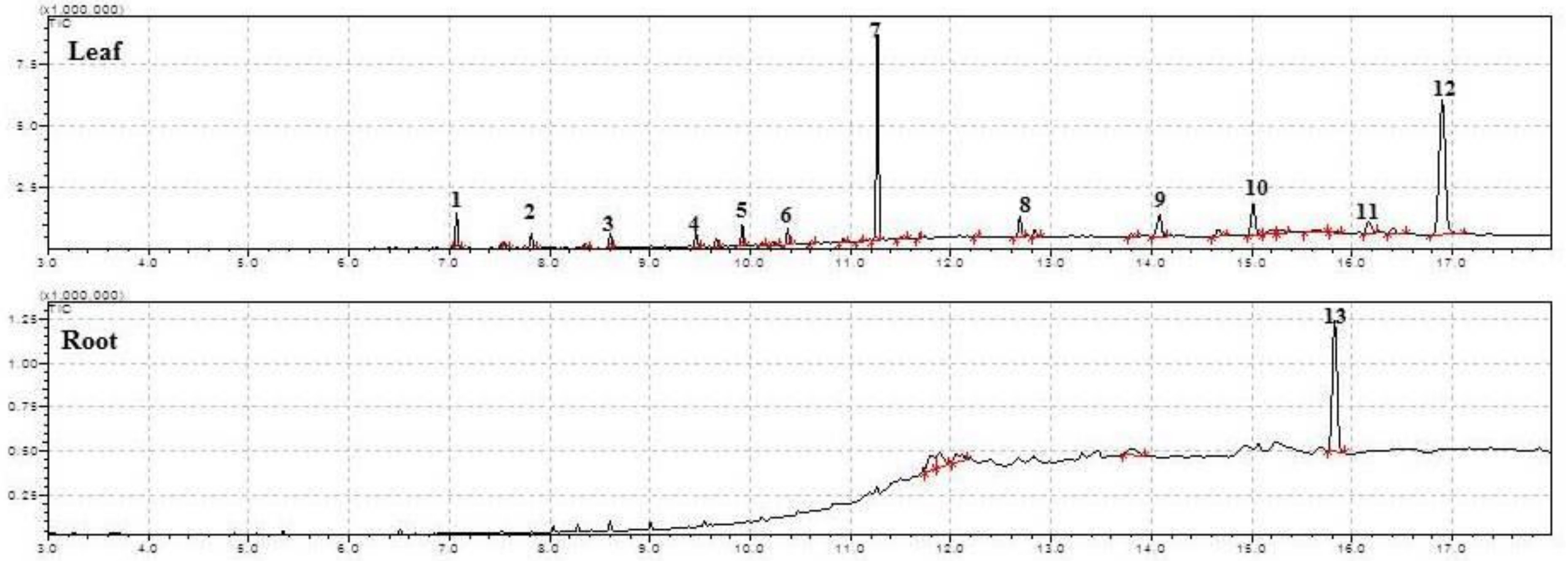


**Figure S3.** Chromatograms of the compounds from *C. clusii* leaves and roots extracted with methanol analyzed by GC-MS. Only the biggest peaks are represented: (1) Bornyl acetate; (2) Linderol; (3) Ionene; (4) Caryophyllene oxide; (5) Viridiflorol; (6) Cubenol; (7) Manoyl oxide; (8) 1-Bromotriacontane; (9) n-Tetratetracontane; (10) Oxomanoyl oxide; (11) Rhododendrol; (12) Pregnane-3,17,20-triol, (3 alpha-5 beta, 20S); (13) 2,3-Oxidosqualene.

**Table S4.** List of compounds extracted from *C. clusii* leaves and roots. The molecular formula, molecular weight, the nature of the compound, the retention time, and the peak area are shown for each compound.

| Plant part | Name of the compound | Formula | Mol. weight | Nature of the compound | Ret. time | Peak area |
|---|---|---|---|---|---|---|
| **Leaf** | Bornyl acetate | $C_{12}H_{20}O_2$ | 196 | Terpene | 7.1 | 490,915 |
| | Linderol | $C_{10}H_{18}O$ | 154 | Terpene | 7.8 | 382,273 |
| | Ionene | $C_{13}H_{18}$ | 174 | Terpene | 8.6 | 276,425 |
| | Caryophyllene oxide | $C_{15}H_{24}O$ | 220 | Terpene | 9.5 | 63,540 |
| | Viridiflorol | $C_{15}H_{26}O$ | 222 | Terpene | 9.9 | 82,184 |
| | Cubenol | $C_{15}H_{26}O$ | 222 | Terpene | 10.4 | 85,235 |
| | Manoyl oxide | $C_{20}H_{34}O$ | 290 | Terpene | 11.3 | 1,227,866 |
| | 1-Bromotriacontane | $C_{30}H_{61}Br$ | 500 | Haloalkane | 12.7 | 178,859 |
| | n-Tetratetracontane | $C_{44}H_{90}$ | 618 | Aliphatic alkane | 14.1 | 295,447 |
| | Oxomanoyl oxide | $C_{20}H_{32}O_2$ | 304 | Terpene | 15.0 | 316,269 |
| | Rhododendrol | $C_{10}H_{14}O_2$ | 166 | Phenolic compound | 16.2 | 376,334 |
| | Pregnane-3,17,20-triol, (3α, 5β, 20S) | $C_{21}H_{36}O_3$ | 336 | Steroid | 16.9 | 1,716,398 |
| **Root** | 2,3-Oxidosqualene | $C_{30}H_{50}O$ | 426 | Terpene | 15.8 | 837,645 |

**Table S5.** Potential phytotoxic effects of the compounds extracted from *C. clusii* leaves and roots. Phytotoxic effects are not attributable to the compound in isolation, but to the extracts containing the compound.

| Name of the compound | Potential phytotoxic effects | Found in | References |
|---|---|---|---|
| Bornyl acetate | Inhibit germination and control seedling growth | *Cistus ladanifer* L. | Verdeguer et al. (2012) |
| Linderol | Not tested | *Thymus vulgaris* L. | TSAI et al. (2011) |
| Ionene | Affect germination and initial radical elongation | *Salvia multicaulis* Vahl. var. *simplicifolia* Boiss. | Mancini et al. (2009) |
| Caryophyllene oxide | Inhibit radicle elongation | *Anisomeles indica* (L.) Kuntze | Batish et al. (2012) |
| Viridiflorol | Inhibit germination and control seedling growth | *Cistus ladanifer* L. | Verdeguer et al. (2012) |
| Cubenol | Inhibit germination and growth of the roots | *Schinus molle* L. | Simionatto et al. (2011) |
| Manoyl oxide | Not tested | *Cistus salvifolius* L., *C. creticus* L. | Mastino et al. (2017) |
| 1-Bromotriacontane | Not tested | *Jatropha curcas* L. | Mahalakshmi et al. (2016) |
| n-Tetratetracontane | Not tested | *Leea indica* (Burm. f.) Merr. | Srinivasan et al. (2008) |
| Oxomanoyl oxide | Not tested | *Pinus banksiana* Lamb. | Conner and Rowe (1977) |
| Rhododendrol | Inhibit germination and seedling growth | *Rhododendron catawbiense* Michx., *R. maximum* L. | Gant (1978) |
| Pregnane-3,17,20-triol, (3α, 5β, 20S) | - | - | - |
| 2,3-Oxidosqualene | Not tested | *Arabidopsis thaliana* (L.) Heynh. | Husselstein–Muller et al. (2001) |

## References


Batish DR, Singh HP, Kaur M, Kohli RK, Singh S (2012) Chemical characterization and phytotoxicity of volatile essential oil from leaves of *Anisomeles indica* (*Lamiaceae*). *Biochemical Systematics and Ecology* **41**:104–109

Conner AH, Rowe JW (1977) New neutral diterpenes from southern pine tall oil. *Phytochemistry* **16**:1777–1781

Gant R (1978) *The role of allelopathic interference in the maintenance of southern Appalachian Heath Balds*. Doctoral Dissertations

Husselstein–Muller T, Schaller H, Benveniste P (2001) Molecular cloning and expression in yeast of 2,3–oxidosqualene– triterpenoid cyclases from *Arabidopsis thaliana*. *Plant Molecular Biology* **45**:75–92

Mahalakshmi R, Eganathan P, Parida AK (2016) Essential oil composition from seedlings of *Jatropha curcas* L. *Journal of Essential Oil Bearing Plants* **19**:421–432

Mancini E, Arnold NA, De Martino L, De Feo V, Formisano C, Rigano D, Senatore F (2009) Chemical composition and phytotoxic effects of essential oils of *Salvia hierosolymitana* Boiss. and *Salvia multicaulis* Vahl. var. *simplicifolia* Boiss. Growing Wild in Lebanon. *Molecules* **14**:4725–4736

Mastino PM, Marchetti M, Costa J, Usai M (2017) Comparison of essential oils from *Cistus* species growing in Sardinia. *Natural Product Research* **31**:299–307

Simionatto E, Chagas MO, Peres MTLP, Hess SC, Silva CB da, Ré-Poppi N, Gebara SS, Corsino J, Morel AF, Stuker CZ, Matos M de FC, Carvalho JE de (2011) Chemical composition and biological activities of leaves essential oil from *Schinus molle* (*Anacardiaceae*). *Journal of Essential Oil Bearing Plants* **14**:590–599

Sokmen A, Jones BM, Erturk M (1999) The in vitro antibacterial activity of Turkish medicinal plants. *Journal of Ethnopharmacology* **67**:79–86

Srinivasan G, Ranjith C, Vijayan K (2008) Identification of chemical compounds from the leaves of *Leea indica*. *Acta Pharmaceutica* **58**:207–214

TSAI M-L, LIN C-C, LIN W-C, YANG C-H (2011) Antimicrobial, antioxidant, and anti-inflammatory activities of essential oils from five selected herbs. *Bioscience, Biotechnology, and Biochemistry* **75**:1977–1983

Verdeguer M, Blázquez MA, Boira H (2012) Chemical composition and herbicidal activity of the essential oil from a *Cistus ladanifer* L. population from Spain. *Natural Product Research* **26**:1602–1609
